# Discharge to home from a palliative care unit: impact on survival and factors associated with home death after the discharge: a cohort study

**DOI:** 10.1186/s12904-023-01314-1

**Published:** 2023-11-30

**Authors:** Nozomu Murakami, Shinya Kajiura, Kouichi Tanabe, Kenichiro Tsukada, Kazuhiko Shibata, Yoshio Minabe, Tatsuya Morita, Ryuji Hayashi

**Affiliations:** 1 Department of Palliative Care Center, Kouseiren Takaoka Hospital, Toyama, Japan; 2 Department of Clinical Oncology, Toyama University Hospital, 2630 Sugitani, Toyama, Toyama Prefecture 930-0194 Japan; 3 Drug Informatics, Faculty of Pharmacy, Meijo University, Nagoya, Japan; 4 Department of Palliative and Supportive Care, Seirei Mikatahara General Hospital, Hamamatsu, Japan

**Keywords:** Palliative care units, Cohort study, Palliative prognostic index

## Abstract

**Background:**

Staying at home during the dying process is important for many patients; and palliative care units (PCUs) can help facilitate home death. This study compared patient survival between those who were discharged to home from a palliative care unit and those who were not, and aimed to identify the factors associated with home death after the discharge.

**Methods:**

This retrospective cohort study used a database of patients admitted to a palliative care unit at Kouseiren Takaoka Hospital in Japan. All consecutive patients admitted to the hospital’s PCU between October 2016 and March 2020 were enrolled. Patient survival and factors potentially associated with survival and place of death were obtained. A total of 443 patients with cancer were analyzed, and 167 patients were discharged to home and 276 were not.

**Results:**

Propensity score matching analyses revealed that median survival time was significantly longer in patients who were discharged to home than those who were not (57 vs. 27 days, *P* < 0.001). Multiple logistic regression analysis identified that worse Palliative Prognostic Index (odds ratio [OR] = 1.21, 95% confidence interval [CI] = 1.03–1.44, *p* = 0.025) and family members’ desire for home death (OR = 6.30, 95% CI = 2.32–17.1, *p* < 0.001) were significantly associated with home death after their discharge.

**Conclusions:**

Discharge to home from palliative care units might have some positive impacts on patient survival.

## Background

Many terminal-stage parients with cancerwish to be cared for at home. Patients who died at home experienced better quality of death than those who died at acute care hospitals [1–3]. Empirical studies, however, indicate that a considerable number of patients actually die at places other than their own home [4–9].

In Japan, although approximately 50% of the public report a desire to receive care at home if diagnosed with terminal cancer [10, 11], the percentage of patients with cancer who actually die at home is as low as approximately 10%, indicating that the wishes of many patients remain unfulfilled. Thus, the FY2018 Revision of Medical Fees of the Ministry of Health, Welfare and Labor clearly stated that certified palliative care units (PCUs) should facilitate home death [11]. From 2018, a minimum discharge-to-home rate of 15% was required for higher hospitalization fees for national insurance coverage to PCU, and many PCUs have made efforts to discharge their patients to home and receive quality end-of-life care there.

Impact of discharge on patient survival is one of the most common concerns among patients and families and can become a barrier for discharge to home. Thus, some studies compared patient survival at home vs. PCU [12–15]. Till date, empirical data have suggested that patient survival could be better in patients who died at home than those who died at hospitals [12–15]. However, the participants in those studies are heterogenous; that is, not limited to patients once admitted to PCUs. No existing research has yet focused on a homogeneous sample of patients admitted to PCUs [12–15]. It might be that discharge from the hospital even once contributes to a prolonged prognosis. Therefore, we designed a study to determine whether discharge from the PCU, even once, is associated with a longer prognosis and aimed to identify the factors associated with home death after the discharge. If discharge from the hospital even once contributes to a longer prognosis, then this might be the greatest contribution of this study, as it provides an incentive to actively encourage discharge in actual clinical practice.

## Methods

This is a retrospective cohort study using a prospectively-collected database of patients admitted to the PCU at Kouseiren Takaoka Hospital, Toyama prefecture in Japan. Data were obtained from the electronic medical records. The hospital was an acute hospital with 533 beds, and the PCU has 16-beds and provides an active home support services in addition to end-of-life care [16, 17]. We chose to examine this study at our institution and with all eligible cases during the time period covered, rather than selecting a sample size in terms of power. This study was conducted with the approval of the Clinical Research Ethics Review Committee of the Kouseiren Takaoka Hospital (Approval No.: #20,190,829,003). We obtained informed consent from all participants.

### Participants

All consecutive patients admitted to the PCU between October 2016 and March 2020 were eligible for this study. No case exclusion criteria were established for this study.

### Measurement variables

On the basis of literature reviews [12–15, 18–21], variables potentially associated with survival and place of death were extracted from the medical records: patient age, sex, primary tumor sites, length of hospital stay, presence or absence of metastases, Palliative Prognostic Index (PPI) [22], symptoms, vital signs (i.e., systolic blood pressure, pulse rate, and SpO_2_), opioid dose (oral morphine equivalent), marital status, the number of co-habiting family members (including patient), presence or absence of a daytime caregiver, whether the primary caregiver was a spouse, family members’ preferred place of care, and family members’ preferred location of death. Further, calorie intake on the first day and presence/absence of delirium within three days of admission were recorded. Primary tumor sites were classified into hepatobiliary pancreatic cancer, respiratory cancer, gastrointestinal cancer, head and neck cancer, urologic cancer, skin cancer, gynecologic cancer, and others. The symptoms were classified as pain, fatigue, dyspnea, disturbance of consciousness, nausea and vomiting, anorexia, abdominal distention, and others.

### Outcomes

Patient survival was defined as the periods from the day of admission to the PCU to death. Each patient was followed up to seven months. Place of death was also recorded.

### Analysis

Patients who were discharged to home from the PCUs and were treated at home for at least one day, were grouped into the discharge-to-home group, and those who were treated in the PCU from admission until death were grouped into the PCU care group.

For comparisons of survival, propensity score matching was estimated using a logistic regression model adjusted for age, sex, PPI, and cancer type. Propensity score matching was implemented using a nearest neighbor matching approach without replacement, with a caliper of 0.04 for optimal precision. Standardized differences were employed as a metric to assess the balance achieved through the matching process. Kaplan‒Meier curves and log-rank tests were utilized to compare survival between the PCU care group and the discharge-to-home group. To evaluate the Kaplan‒Meier curves after propensity score matching, curves were drawn for patients before propensity score matching as a sensitivity analysis. To analyze the factors affecting survival, a Cox proportional hazards model was used, and hazard ratios (HRs) and 95% CIs were calculated.

The primary outcome measure for this study is whether there is a significant difference in survival after propensity score matching. The following secondary outcome measures are also to be evaluated. To identify the factors associated with death at home after discharge, patient backgrounds were compared between the patients who were discharged to home and eventually died at home and those who were discharged to home but eventually died at the hospital (PCU). Comparisons were performed using Student’s t-test or χ2 test wherever appropriate. Multiple logistic regression analysis was performed as a form of multivariate analysis to investigate factors affecting the place of death remaining in the final model, and HRs and 95% CIs were calculated.

The significance level was set at 5%, and all analyses were conducted using IBM SPSS Statistics ver. 27 (IBM Corporation).

## Results

### Patient backgrounds

**Fig. 1:**
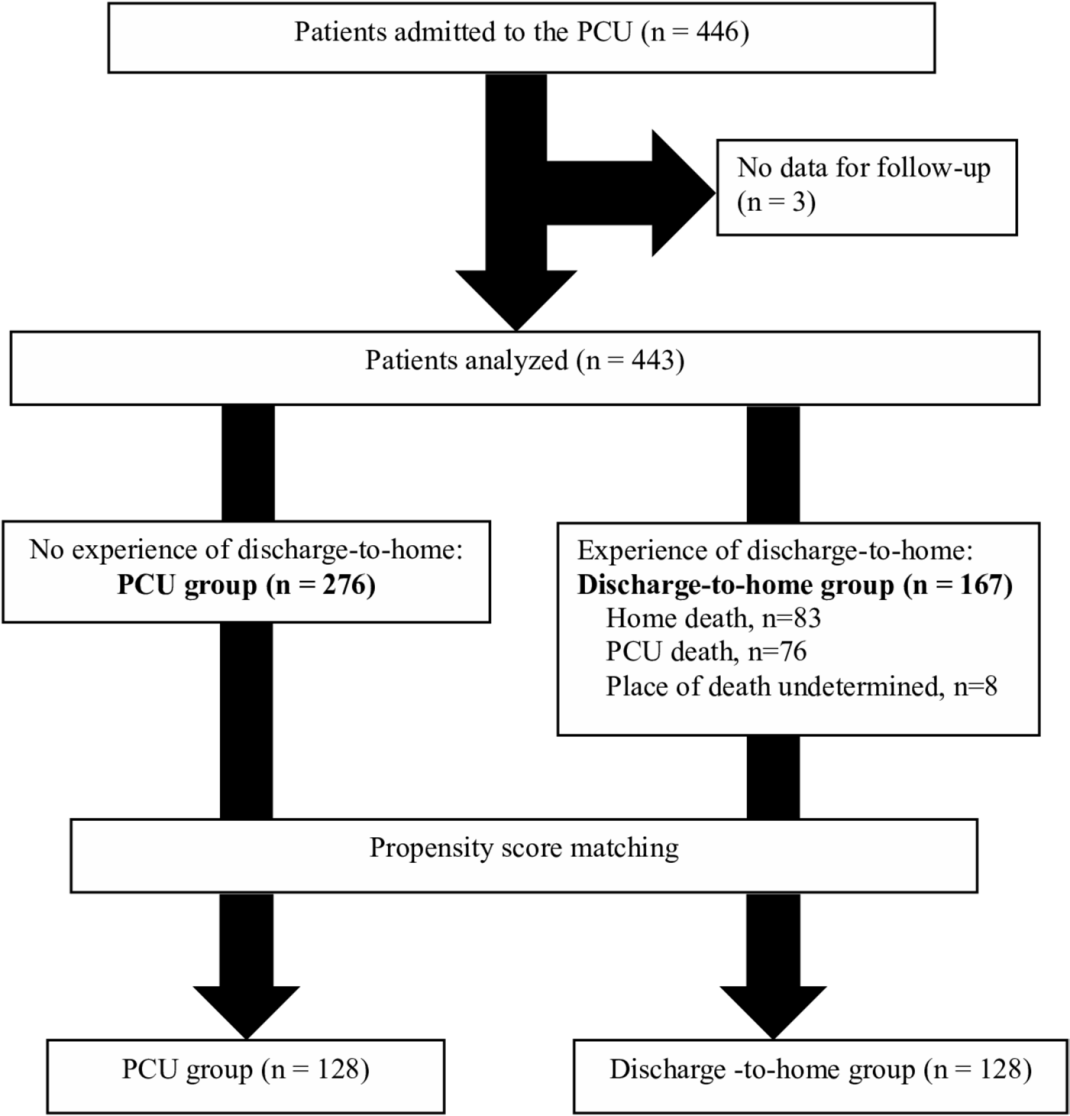
Patient flow. PCU: palliative care unit

During the study period, a total of 446 patients with cancer admitted to the PCU were enrolled. Three patients with no follow-up data were excluded, resulting in a total of 443 patients for analysis (Fig. 1). Of them, 167 were in the discharge-to-home group and 276 in the PCU group. Patients who were discharged to home were found to be in a significantly better general condition measured by the PPI at admission (Table 1, left column).

**Table 1:** Patient backgrounds before and after propensity score matching

	Before matching				After matching			
	PCU care (*n *= 276)	Discharge-to-home (*n* = 167)	*P*-value	Standardized difference	PCU care (*n* = 128)	Discharge -to-home (*n* = 128)	*P*-value	Standardized difference
Age (in years), mean (SD)	72.96 (10.68)	73.46 (11.26)	0.637	0.046	73.96 (10.45)	73.50 (11.49)	0.737	0.042
Sex (male/female)	161/115	92/75	0.504	0.065	74/54	69/59	0.529	0.079
PPI at admission, mean (SD)	6.2 (3.22)	3.50 (2.77)	**< 0.001**	0.902	4.20 (2.70)	4.19 (2.70)	0.982	0.004
Hepatobiliary pancreatic cancer	50 (18.1%)	25 (15.0%)	0.392	0.083	23 (18.0%)	18 (14.1%)	0.394	0.10
Respiratory cancer	75 (27.2%)	41 (24.6%)	0.543	0.059	28 (21.9%)	31 (24.2%)	0.656	0.055
Gastrointestinal cancer	80 (29.0%)	41 (24.6%)	0.310	0.099	31 (24.2%)	35 (27.3%)	0.568	0.071
Head and neck cancer	12 (4.3%)	19 (11.4%)	**0.005**	0.26	11 (8.6%)	8 (6.3%)	0.474	0.088
Urological cancer	25 (9.1%)	19 (11.4%)	0.429	0.076	19 (14.8%)	18 (14.1%)	0.859	0.020
Skin cancer	4 (1.4%)	3 (1.8%)	0.530	0.032	1 (0.8%)	1 (0.8%)	1.0	0
Gynecological cancer	23 (8.3%)	14 (8.4%)	0.985	0.004	13 (10.2%)	13 (10.2%)	1.0	0
Others	7 (2.5%)	5 (3.0%)	0.774	0.031	2 (1.6%)	4 (3.1%)	0.342	0.099

*PCU *Palliative care unit, *PPI *Palliative prognostic index

### Comparisons of patient survivals

Propensity score matching was performed to assess the backgrounds of patients from the two groups, which resulted in 128 matched cases in both groups (Table 1, right column).

**Fig. 2:**
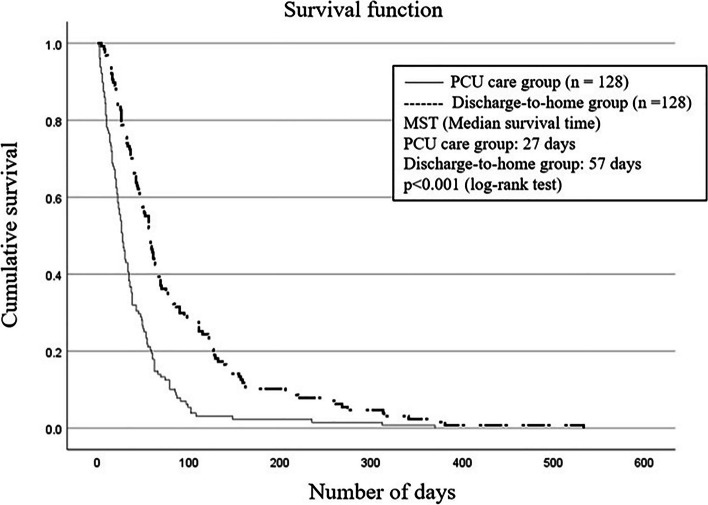
Kaplan-Meier curve after propensity score matching.  PCU: palliative care unit

As shown in Fig. 2, a comparison of survival after propensity score matching revealed that the median survival time (MST) for the discharge-to-home group was 57 days, while the MST for the PCU care group was 27 days, with the discharge-to-home group having significantly longer survival than the PCU care group (p < 0.001). The Cox proportional hazards model also showed that place of care had a significant impact on survival. Other factors significantly associated with survival included; length of hospital stay, pulse rate, co-habiting family members, pain, and PPI at admission (data not shown).

### Factors associated with home death after patients were discharged to home from PCU

Among 167 patients of discharge-to-home group, eight were still alive after the seven-month follow-up period and were thus, excluded from this analysis (Fig. 1). Of the remaining 159 patients, 76 were eventually readmitted to the PCU and died there (PCU death group); and 83 patients received end-of-life care at home until death (Home death group).

Compared with the PCU death group, patients of home death group were significantly older, had shorter admission periods, had worse PPI at admission, had a daytime caregiver, and that family’s preferred place of death was home (Table 2).

**Table 2:** Comparisons of patients who died at PCU and those who died at home after discharge: univariate analyses

	PCU death group (*n* = 76)	Home death group (*n* = 83)	*p*-value
Age (in years), mean (SD)	71.4 (11.2)	75.3 (11.4)	**0.031**
Sex (male/female)			
Male	42 (55.3%)	43 (51.8%)	0.751
Female	34 (44.7%)	40 (48.2%)	
Number of days of hospital stay, Mean (SD)	28.5 (17.1)	21.7 (14.1)	**0.007**
Presence of metastases			
Present	51 (67.1%)	58 (69.9%)	0.864
Absent	24 (35.8%)	25 (30.1%)	
PPI on admission, mean (SD)	2.6 (2.1)	4.5 (3.0)	**< 0.001**
Systolic blood pressure, mean (SD)	115.8 (18.7)	115.4 (20.7)	0.885
Pulse rate, mean (SD)	85.3 (16.0)	82.9 (17.0)	0.363
SpO_2_, mean (SD)	97.0 (2.0)	96.6 (2.0)	0.216
Calorie intake on first day, mean (SD)	978.6 (567.1)	828.0 (583.3)	0.101
Opioid dose (oral morphine conversion, mg/day), mean (SD)	38.1 (66.3)	28.3 (45.5)	0.275
Delirium within 3 days of admission	5 (6.5%)	13 (15.7%)	0.083
Marital status			
Married	70 (92.1%)	82 (98.8%)	0.055
Single	6 (7.9%)	1 (1.2%)	
Co-habiting family			
Alone	12 (15.8%)	5 (6.0%)	0.070
Two or more people	64 (84.2%)	78 (94.0%)	
Daytime caregiver	43 (56.5%)	65 (78.3%)	**0.002**
Primary caregiver			
Spouse	35 (46.1%)	34 (41.0%)	0.522
Other	40 (52.6%)	49 (59.0%)	
Family’s preferred place of care			
Hospital	8 (10.5%)	6 (7.2%)	0.576
Home	64 (84.2%)	76 (91.6%)	
Family’s preferred place of death			
Hospital	38 (50.0%)	20 (24.1%)	**< 0.001**
Home	16 (21.1%)	52 (62.7%)	
Primary tumor sites			0.146
Hepatobiliary pancreatic cancer	11 (14.5%)	14 (16.9%)	
Respiratory cancer	13 (17.1%)	24 (28.9%)	
Gastrointestinal cancer	10 (23.7%)	21 (25.3%)	
Head and neck cancer	13 (17.1%)	4 (4.8%)	
Urological cancer	8 (10.5%)	11 (10.5%)	
Skin cancer	3 (3.9%)	0 (0.0%)	
Gynecological cancer	7 (9.2%)	7 (8.4%)	
Others	3 (3.9%)	2 (2.4%)	
Major symptoms			0.259
Pain	43 (56.6%)	41 (49.4%)	
Fatigue	10 (13.2%)	13 (15.7%)	
Dyspnea	6 (7.9%)	15 (18.1%)	
Disturbed consciousness	3 (3.9%)	4 (4.8%)	
Nausea and vomiting	3 (3.9%)	2 (2.4%)	
Anorexia	3 (3.9%)	5 (6.0%)	
Abdominal distension	4 (5.3%)	0 (0.0%)	
Other	4 (5.3%)	3 (3.6%)	

*PCU *Palliative care unit, Continuous values were expressed as mean and standard deviations

As shown in Table 3, multiple logistic regression analysis identified that the significant factors for home death after discharge were PPI at admission (OR = 1.219, 95% CI = 1.026–1.448, *p* = 0.025) and family members’ preference of home as place of death (OR = 6.297, 95% CI = 2.322, 17.075, *p* < 0.001).

**Table 3:** Multiple logistic regression analysis about factors associated with home death after the discharge

	Partial regression coefficient	Odds ratio	95% confidence interval		*p*-value
			Upper limit	Lower limit	
PPI on admission	0.198	**1.219**	1.026	1.448	**0.025**
Pulse rate	− 0.031	0.969	0.939	1.001	0.054
Presence of a daytime caregiver	1.468	4.340	0.942	19.988	0.060
Family’s preferred place of death was home	1.840	**6.297**	2.322	17.075	**< 0.001**

*PPI *Palliative prognostic index

## Discussion

This study compared survival of patients who were discharged to home from PCU and those who were not, and we explored factors associated with home death after the discharge.

One of the most important findings was that patient survival was significantly longer in patients who experienced discharge-to-home than in those who did not, after adjusting for factors that could have influenced prognosis and place of death. Although the exact mechanism for this is difficult to ascertain, the findings are consistent with other empirical studies on more heterogenous population [12–15]. For example, Hamano et al. compared survival time of patients with cancer who died at home with those who died in the hospitals [14, 15]. The average survival time for patients whose life expectancy was predicted to be days based on the PiPS models was 13 days at home compared with nine days at the hospitals; for those expected to live for weeks, the survival time was 36 days at home and 29 days at the hospitals [14]. Interestingly, patients who died at home received less burdensome medical interventions, such as administration of IV fluids in the 2‒3 days preceding death and antibiotics within three weeks before their death [14]. The longer survival span in patients at home was confirmed after adjustment of medical treatment they received in another cohort [15]. Other study group also compared survival periods between two groups of patients with cancer treated by the palliative care team of a single cancer hospital, and revealed a significantly longer survival in the home-care group [13]. The authors also reported that the use of the Home Palliative Care Regional Coordination Pass as an information-sharing tool was significantly associated with longer length of home care　 [16, 17]. These findings suggest that home-based care may not only improve the quality of life but might also contribute to longer survival.

Second important finding was the clear identification of factors significantly associated with home death in patients who were discharged to home from a PCU. In this study, the most influential factor of preference for home death was the family’s preference during the event of home death. The ability to care for the patient at home could also have a significant impact on supporting home care. These findings are consistent with previous studies [18–22], and they confirm that opinions of family members and availability of a daytime caregiving service are important factors influencing home death for patients who were discharged to home. The average performance status was 2.67 and PPI was 3.48 for patients at the time of admission to the PCU. This implies that patients who were discharged to home spent more than half of the day in bed and had very limited prognosis, and thus families have an important role in the decision to return home.

The strengths of this study included obtaining a homogenous sample of patients who were discharged to home from PCU, prospective collection of data as a part of routine practice, use of statistical methods to adjust covariates, and a relatively high number of patients. We also showed that discharge from the hospital, even once, might be related to a prolonged prognosis. These results might suggest a motivation to actively encourage discharge in clinical practice. Showing this data to patients might encourage them to discharge them from the hospital. Contrarily, this study also has some limitations. First, the retrospective nature of the data analyses could cause some bias in measurement variables.　Second, although we used propensity score matching for adjusting patient backgrounds, unmeasured factors were not adjusted, like use of anticancer treatment including chemotherapy, comorbidities, and economic characteristics. Third, we classified the patients based on the outcome (discharge or death), and thus certain patients who had wished to return to home but eventually died in the PCU during the arrangement period were included in the PCU group, which could overestimate survival in the discharge-to-home group. Additionally, no formal sample size calculation was performed. Future research needs to include prospective studies conducted with sufficient sample sizes. However, the ethical issues involved in randomizing patients to discharge home versus continued hospitalization make this practically difficult, and it might be realistic to conduct a multicenter study with similar case groups and use meta-analysis techniques to conduct this study.

## Conclusion

The study confirmed that survival was longer in patients who had been discharged to home from PCU than in patients who were not, after adjusting for factors that might affect prognosis and place of death. Thus, it can be concluded that discharge to home from PCUs might have some positive impact on patient survival.

## Data Availability

The datasets generated and analyzed for this study are available from the corresponding author on reasonable request.

## Abbreviations

CI: Confidence interval
HR: Hazard ratio
MST: Median survival time
PCU: Palliative care unit
PPI: Palliative prognostic index
